# Assessment of nerve regeneration using nerve guidance conduits in inferior alveolar nerve injuries

**DOI:** 10.6026/973206300212988

**Published:** 2025-09-30

**Authors:** Ankita Shrivastava, Manoj Manohar, Kanishka Guru, Mony Behera, Yeso Alekhya, Sameer Gupta

**Affiliations:** 1 Department of Dentistry, Gajra Raja Medical College, Gwalior, Madhya Pradesh, India; 2 Department of Oral and Maxillofacial Surgeon, Corona Dental Care, Vidyaranyapura, Bangalore, India; 3 Kalinga Institute of Dental Sciences (KIDS), Kalinga Institute of Industrial Technology (KIIT) Deemed to be University, Bhubaneswar, Odisha, India; 4 Department of Oral Medicine and Radiology, Meghna Institute of Dental Sciences, Nizamabad, Telangana; 5 Department of Oral & Maxillofacial Surgery, Inderprastha Dental College and Hospital, Sahibabad, Ghaziabad, Uttar Pradesh, India

**Keywords:** Inferior alveolar nerve, nerve regeneration, nerve guidance conduit, peripheral nerve injury, histomorphometry, rabbit model

## Abstract

Injuries to the inferior alveolar nerve (IAN) often cause persistent sensory deficits and current treatment options such as autografts have limitations. Therefore, it is of interest to evaluate the role of collagen-based nerve guidance conduits (NGCs) in promoting IAN regeneration in 20 rabbits with 5 mm nerve gaps. Functional recovery was assessed by whisker movement scores, electrophysiology and histomorphometric analysis at 4, 8 and 12 weeks. Results showed significantly better outcomes in the NGC group, including higher whisker movement scores, improved CMAP amplitudes and greater axon counts compared to controls. Thus, we show that NGCs enhance structural and functional nerve recovery and represent a promising therapeutic approach for peripheral nerve repair in the maxillofacial region.

## Background:

The inferior alveolar nerve (IAN), which is a major branch of the mandibular division of the trigeminal nerve, is frequently at risk of injury during oral and maxillofacial procedures like surgical extraction of deeply impacted third molars, implant insertion and orthognathic surgeries [1]. Damage to this nerve can lead to temporary or permanent sensory deficits (paresthesia, dysesthesia, or a total lack of sensation on the lower lip, chin and gingival area), which is a great concern for a patient's quality of life [2, 3]. Peripheral nerve injuries are most commonly repaired using microsurgical techniques (direct neurorrhaphy or autologous nerve grafting). Nevertheless, such methods have been linked to some shortcomings, such as donor site morbidity, limited supply of grafts and a lack of nerve size match [4, 5]. In recent years, tissue engineering approaches, especially the application of nerve guidance conduits (NGCs), have offered a promising alternative for stimulating peripheral nerve regeneration [6]. NGCs provide a permissive scaffold for axons to promote regeneration and inhibit fibrous ingrowth, thereby facilitating a process of guided nerve repair for short nerve gaps [7]. NGCs have been fabricated using various materials, such as natural biopolymers like collagen, chitosan, or gelatin and synthetic polymers. Among these, collagen-based conduits have shown excellent biocompatibility and biodegradability, making them suitable for clinical use in nerve repair [8, 9]. Investigations involving the use of NGCs as promoters of axonal regeneration and facilitators of functional recovery in facial and sciatic nerve injuries in animals have produced promising results [10].

Recent innovations in biomaterials and tissue engineering have played an important role in developing bio resorbable and biocompatible nerve guidance conduits that are designed for clinical use. Such conduits mimic the natural extracellular matrix (ECM) and can provide structural and biochemical guidance during axonal sprouting and Schwann cell migration [11]. The addition of growth factors, surface enhancement and aligned micro channels in conduits further improves nerve regeneration by directing axons and accelerating reinnervation of the target region [12]. The effectiveness of these interventions in animal models has paved the way for translational applications, with a quest to optimize success in treating patients with peripheral nerve injuries, including those in the craniofacial region. A biomolecule that has been extensively researched for the development of nerve conduits is collagen, a naturally derived ECM protein. It shows an outstanding level of biocompatibility has a low immunogenicity profile and is capable of supporting cell adhesion and proliferation [13]. A number of smaller studies have shown that collagen-based conduits can be as good as, or even better than, autografts for short-gap nerve injuries, especially when they include additional neurotrophic factors or Schwann cells [14]. Therefore, it is of interest to investigate the regenerative capacity of a collagen-based nerve guidance conduit in a rabbit model of IAN nerve injury using a combination of functional, electrophysiological and histological outcome measures.

## Materials and Methods:

## Study design and ethical approval:

This experimental animal study was conducted to evaluate the efficacy of collagen-based nerve guidance conduits (NGCs) in promoting nerve regeneration following inferior alveolar nerve (IAN) injury.

## Animal selection and grouping:

A total of 20 healthy adult male New Zealand white rabbits (weighing 2.5-3.0 kg) were randomly divided into two groups (n=10 per group). Group A served as the control group and underwent IAN transection without further intervention. Group B received a collagen-based nerve guidance conduit following nerve transection to bridge the created nerve gap.

## Surgical procedure:

All surgical procedures were performed under general anesthesia induced by intramuscular injection of ketamine (35 mg/kg) and xylazine (5 mg/kg). The mandibular region was shaved and disinfected. A unilateral incision was made along the inferior border of the mandible to expose the IAN. In both groups, a 5 mm segment of the nerve was excised to create a nerve gap. In Group B, the gap was bridged using a sterile, prehydrated, cylindrical collagen-based conduit (10 mm length, 2 mm inner diameter), with the nerve stumps inserted 2.5 mm into each end of the conduit and secured using 8-0 nylon sutures under microscopic guidance.

## Postoperative care and monitoring:

Postoperative care included administration of analgesics (meloxicam 0.2 mg/kg) and antibiotics (enrofloxacin 10 mg/kg) for three days. Animals were housed individually in standard cages and monitored daily for wound healing, behavioral changes and feeding activity. No signs of infection or wound dehiscence were observed during the study period.

## Functional and electrophysiological assessment:

Functional recovery was assessed using a modified whisker movement scoring system (scale of 0-5) at 4, 8 and 12 weeks postoperatively. Electrophysiological analysis was conducted at 12 weeks under anesthesia. Compound muscle action potentials (CMAPs) were recorded by stimulating the mental nerve and recording from the orbicularis oris muscle using needle electrodes. CMAP amplitude and latency were analyzed using a digital EMG system.

## Histological and morphometric analysis:

At the end of the 12-week period, animals were sacrificed with an overdose of sodium pentobarbital. The nerve segments, including the conduit and adjacent stumps, were harvested and fixed in 10% buffered formalin. Tissues were processed for paraffin embedding, sectioned at 5 µm and stained with hematoxylin-eosin and toluidine blue. Histomorphometric analysis was performed under light microscopy to quantify regenerated axon density, fiber diameter and myelin sheath thickness.

## Statistical analysis:

Data were analyzed using SPSS version 25.0. Results were expressed as mean ± standard deviation. Intergroup comparisons were made using independent sample t-tests and repeated measures ANOVA was used for time-dependent assessments. A p-value of less than 0.05 was considered statistically significant.

## Results:

The whisker movement scores showed progressive improvement in both groups over 12 weeks; however, Group B (NGC-treated) exhibited significantly better recovery at each time point. At week 4, the mean score in Group A was 1.2 ± 0.4, whereas Group B had a mean score of 2.5 ± 0.6. By week 12, Group B achieved a near-complete recovery with an average score of 4.3 ± 0.5 compared to 2.1 ± 0.4 in Group A (p < 0.01) (Table 1 - see PDF). Compound muscle action potential (CMAP) amplitude was markedly higher in the NGC group. At 12 weeks, Group B exhibited a mean CMAP amplitude of 3.2 ± 0.3 mV, while Group A had a significantly lower amplitude of 1.4 ± 0.2 mV (p < 0.001). Latency values were also more favorable in Group B (Table 2 - see PDF). Histological sections showed superior nerve regeneration in Group B, with well-organized nerve fibers and thicker myelin sheaths. The average myelinated axon count in Group B was 850 ± 45, significantly higher than 490 ± 38 in Group A (p < 0.001). Nerve fiber diameter and myelin thickness were also significantly greater in the NGC group, suggesting enhanced maturation and repair (Table 3 - see PDF). As seen in Tables 1-3 (see PDF), all parameters indicated significantly improved outcomes in the NGC-treated group, supporting the effectiveness of collagen-based nerve conduits in enhancing functional and structural recovery of the injured inferior alveolar nerve.

## Discussion:

This study shows that collagen-based nerve guidance conduits (NGCs) can significantly improve functional and structural outcomes in a rabbit model after inferior alveolar nerve (IAN) injury repair. The results are similar to other studies which have expressed the potential use of NGCs as an alternative to autologous nerve grafts in the repair of peripheral nerves [1, 2]. A functional assessment based on whisker movement scoring showed statistically significant and consistent improvement in the NGC group at all-time intervals, which led to the conclusion of successful axonal regeneration and reinnervation. These findings are consistent with previous animal studies based on identical facial nerve models where collagen conduits allowed for better functional recovery than untreated or sutured nerves [3, 4]. These were also proven by electrophysiological analysis, as the CMAP amplitudes were greater and there were shorter latencies in the NGC group compared to the control group, which was an indication of improved conduction and restoration of nerve continuity. It has already been shown in the literature that functional nerve regeneration is associated with the regaining of normal electrophysiology and normalized conduction velocity [5, 6]. Histologically, Group B proved to have a higher density of myelinated axons, a larger fiber diameter and a thicker myelin sheath, indicating greater axonal regrowth and maturation. These results are in line with others who used collagen conduits to regenerate the sciatic and facial nerves, in which the biological microenvironment that the collagen provides promotes the proliferation of Schwann cells, the synthesis of neurotrophic factors and axonal outgrowths [7, 8]. Moreover, the porous nature of collagen allows the infiltration of cells and the deposition of the extracellular matrix, which is essential for successful nerve healing [9]. The great biocompatibility and biodegradability of collagen-based NGCs are a main benefit, as they eliminate the need for additional surgery for removal and reduce foreign body responses [10]. Also, the similarity of collagen to the original components of the extracellular matrix creates favorable conditions for the attachment and orientation of nerve cells [11]. These properties have already been proven in preclinical and preliminary clinical studies related to digital and lingual nerve injuries [12, 13]. The wide applicability of the present study is furthered by the success of its implementation in the maxillofacial area, especially with regard to the healing of IAN defects caused by trauma, tumor removal, or iatrogenic factors. Although autografts are still the clinical standard, they are restricted by donor site morbidity, the presence of neuromas and length mismatch complications [14, 15]. These current conclusions indicate that collagen-based NGCs may provide similar results in small-length nerve gaps, particularly when applied in a cranial form such as the mandible, where aesthetic and functional aspects are critical.

## Conclusion:

The use of collagen-based NGCs for promoting functional and histological regeneration of the inferior alveolar nerve is shown. With further optimization and validation, these conduits could provide a reliable alternative to autografts for peripheral nerve injuries in the orofacial region.
